# Regional inequalities and substitutability of health resources in the Czech Republic: a five methods of evaluation

**DOI:** 10.1186/s12960-021-00630-y

**Published:** 2021-07-17

**Authors:** Martin Dlouhý

**Affiliations:** Faculty of Informatics and Statistics, Prague University of Economics and Business, 4 Winston Churchill Sq., 13067 Prague 3, Czech Republic

**Keywords:** Regional inequality, Inequality measures, Common weights model, Data envelopment analysis, Production frontier model, Czech Republic

## Abstract

**Background:**

An analysis of the regional distribution of health resources is one of the tools for evaluating equal geographic access to health care. The usual analytical approach to an assessment of regional differences is to evaluate each health resource separately. This is a sensible approach, because there may be systematic reasons for any differences, for example, higher salaries in urban areas. However, a separate evaluation of the regional distribution of health resource capacities may be misleading. We should evaluate all health resource capacities as a whole and consider the substitutability of resources.

**Objective:**

This study aims to measure regional inequalities in the Czech Republic with the help of alternative approaches to the evaluation of regional inequalities in the case of several substitutable health resources.

**Methods:**

Five alternative evaluation methods (models) are described and applied: the separate evaluation, expert model, market model, common weights model, and production frontier model.

**Results:**

The regional distribution of physicians and nurses in the Czech Republic in 2017 was evaluated. In spite of many regulations at the national and regional levels, we have found inequalities in regional resource distribution. The models that consider all health resources and the possibility of a resource substitution show lower inequalities between regional health resource capacities.

**Conclusion:**

Both researchers and policy-makers should always consider the possibility of resource substitutions in the assessment of regional inequalities.

## Background

Equality is an important goal of health policy, and this goal strongly affects the financing and organization of the health system [1]. Unequal regional distribution of health resources and their unequal utilization have been described by studies from many countries [2–7] even though the majority of health care is funded from public sources (taxes or public health insurance) and the provision of health care is highly regulated. One of the findings of clinical practice variation research is that regional variations in health care utilization and spending are systematic (not just random noise), substantial, pervasive and persistent over time [6]. Hence, any regional inequalities in health resource capacities that do not reflect local health needs can be seen as a failure of national health policy.

However, unequal distribution of health resources is not only an issue of equality, but also of efficiency. Let us have regions *A* and *B* that have populations with the same health needs, but region *A* has more physicians per capita than *B*. There is an oversupply of physicians in *A* and an undersupply in *B*. If marginal social benefits from services are decreasing, then the unequal distribution of physicians cannot be efficient. Patients in region *A* gain lower marginal social benefits than patients in region *B* could gain. Hence, the total social benefit is lower than it could be. In such a situation, health policy with the objective of equal resource distribution also increases the efficiency of the health system.

The objective of this study is to evaluate regional inequalities of health resources in the Czech Republic. The country is administratively divided into 14 regions. Prague, the capital, has the status of a region. In the Czech Republic, health services are reimbursed by public health insurance, which should guarantee equal access to health services for the whole population.

## Methods

An analysis of the regional distribution of health resources is one of the tools for evaluating equal access to health care. The usual analytical approach to an assessment of regional differences is to evaluate each health resource separately. We argue that the separate evaluation of health resource capacities may be misleading. We should evaluate health resource capacities as a whole, and we should consider the possibility of substitution of resources. This approach will give us more accurate information on total resource capacity in a region and on the extent of regional inequalities.

This paper is, in particular, focused on the substitution between physicians and nurses, which is quite a discussed issue in the literature. For example, the so-called Wanless Report [8] states that nurse practitioners could undertake at least 20 per cent of the work of physicians while maintaining the safety and quality of care. Research evidence shows that nurse practitioner consultations are longer, so there is the need of 1.5 nurse practitioners as a substitute for one physician. Laurant et al. [9] suggest in their review that trained nurses can provide as high-quality care as primary care physicians and achieve as good health outcomes for patients. According to the review article by Fulton et al. [10], there is a piece of substantial evidence that task shifting is an important policy option to help alleviate workforce shortages and skill-mix imbalances. In their review article, Buchan and Dal Poz [11] state that the evidence on the doctor/nurse relationship indicates an unrealized scope for extending the use of nursing staff and for the development of care delivery led by nurses/midwives. Martinez-Gonzales et al. [12] find that trained nurses appeared to be better than physicians in some selected clinical parameters, but there is insufficient evidence on the outcomes of some other parameters.

The issue of substitution is also closely related to the scope of practice and the model of care. The Report of the Canadian Academy of Health Sciences [13] identifies micro, meso, and macro levels of barriers and enablers that influence the optimization of health care professional scopes of practice and supportive models of care. Such health policy analysis is needed for managing changes in scopes of practice.

In this study, we present alternative approaches to the evaluation of regional inequalities in the case of substitutable health resources. We will show the application of the described methods on the example of regional inequalities from the Czech Republic.

As a first look at the data, separate evaluation is a sensible approach, because there may be systematic reasons for any differences, for example higher salaries in urban areas. However, separate evaluation of each health resource is the proper evaluation method if it is believed that resource substitution is impossible. Unlike the method of separate evaluation, the other methods presented below assume that health resources are, at least to some extent, substitutes. It is assumed that the lower health resource capacities in one resource category can be compensated by higher capacities in another resource category. In such a case, the size of regional inequality is expected to be lesser than the inequality calculated by a separate evaluation. In the case of several health resources, the total health resource capacity can be obtained by the weighted sum of individual health resource capacities. A comparison of health resource capacities in this situation is a problem of multiple criteria decision-making, specifically of the weighted sum model. The critical issue of the weighted sum model is the setting of relative resource weights. The question which is addressed in this study is how the values of these resource weights can be obtained. A survey among experts who can give us their subjective views is one possibility (here called the expert model). An alternative option is calculating resource weights objectively by a mathematical model (common weights model, production frontier model). To use the prices of health resources is the last possibility (here called the market model).

### Separate evaluation

The most common method of inequality evaluation of regional resource capacities found in the literature is the separate evaluation of each health resource. The researchers usually compare regional capacities of physicians, nurses, hospital beds and the numbers of medical technology. They evaluated only a single health resource or no substitution among multiple health resources was considered [14–21]. In such cases, the total sum of health resource capacities was not calculated, and inequality measurement could be misleading.

Separate evaluation of each health resource is simple and easily understandable by all health policy stakeholders, which is surely an advantage. However, separate evaluation has a serious disadvantage in ignoring possible substitutions among health resources. For simplicity, let us assume that more is better, although it is not always the case. Hence the population in region *A* has surely better access to health care than the population in region *B* only in the case if region *A* mathematically dominates region *B*, i.e. capacities of all health resources in region *A* are higher than the capacities in region *B*. In the case that some capacities of health resources are higher in region *A* and some capacities are higher in region *B*, the separate evaluation method is not able to decide which regional population is better-off.

### Expert model

In the expert model, we believe in the knowledge of experts that is based on the accumulated professional experience. Some form of a questionnaire is usually used in order to obtain necessary input data from a panel of experts for setting the resource weights. Because the task of weight setting is not easy, we recommend the Delphi method, which is essentially an iterative survey that provides feedback from the participating experts over successive rounds. The aim of the method is to reach a consensus as an expert reviews his or her initial opinion in the face of information from other experts [22]. Another possible tool is the Sheffield elicitation framework (SHELF), which is a method of capturing expert knowledge about uncertain quantities in the form of a probability distribution [22, 23]. The choice of the expert panel and a definition of an expert are crucial in any method. We describe an expert as a person with sufficient professional experience in a managerial position at the hospital, health insurance fund, Ministry of Health.

### Market model

We assume that prices are relevant signals, so in the market (economic) model, the weights of health resources (production factors) are estimated by their market prices. However, we have to admit that the prices of production factors are heavily regulated in the health system. In the market model, the resource weights are set as average monthly salaries. The ratio of salaries shows how many times a physician is more productive than a nurse. An alternative possibility is to apply regional prices (salaries) that can form a sub-category named as the local market model. In this case, substitution between resources is allowed to be different in each region.

### Common weights model

The common weights model estimates the optimal resource weights from the regional data. The idea of the model is that the national health system as a whole tends to maximize the value of total health resource capacity. The optimal relative ratios of resource weights are the same for all regions in the given country. The objective function of the common weights model is a maximization of the total weighted sum of health resource capacities and the resource weights are the variables. Let us have a country with *n* regions that use *m* inputs to serve the regional population. In this case, we assume that the regional population is the single output that can be omitted from the model if the inputs are expressed in the numbers per inhabitant. The mathematical formulation of the common weights model is:

1 $$\begin{gathered} {\text{Maximize}} \mathop \sum \limits_{j = 1}^{n} \,\varphi_{j} \hfill \\ {\text{subject to }} \varphi_{j} = \mathop \sum \limits_{i = 1}^{m} v_{i} x_{ij},\, j = 1, 2, \ldots ., n, \hfill \\ \mathop \sum \limits_{i = 1}^{m} v_{i} x_{ij} \le 1,\, j = 1, 2, \ldots ., n, \hfill \\ v_{i} \ge \varepsilon ,\, i = 1, 2, \ldots , m, \hfill \\ \end{gathered}$$

where *φ*_*j*_ is the normalized capacity score of the region *j* (the score is set to be 1 or 100 for the region with the highest total capacities and is lower for the others), *x*_*ij*_ is the capacity of health resource *i* per 1000 inhabitants in region *j*, *ε* represents an infinitesimal constant that assures that the weight for each health resource is greater than zero. The resource weights *v*_*i*_ are variables in the common weights model.

### Production frontier model

The production frontier model estimates the optimal resource weights from the regional data. The production frontier is a more flexible approach of resource weights estimation than the common weights model, because the optimal weights of health resources can differ for each region. In the production frontier model, the health resources can be considered as inputs, and the served population (as a basic measure of health need) is the single output. In this case, the production function is estimated by the “efficient” units with minimal amounts of resources. Thus, the capacities of “inefficient” units are compared to the most efficient production frontier. In the second form of the production frontier, the regional population is modelled as the single input and health resources are model outputs. In this alternative that will be used in this study, the production frontier is estimated by the most “inefficient” units, i.e. by the units with the maximal amount of resources.

The production frontier can be estimated by the stochastic frontier analysis, which is an econometric method [24], or by data envelopment analysis (DEA), which is a method based on the mathematical programming [25, 26]. In this study, we will use DEA to estimate the production frontier.

DEA constructs the production frontier and evaluates the technical efficiency of units. DEA is based on the mathematical programming and estimates the production frontier as the piecewise linear envelopment of the data. The unit uses the set of inputs to produce the set of outputs. The technical efficiency of the production unit is defined as the ratio of its total weighted output to its total weighted input or vice versa. In the DEA model, each unit sets its input and output weights to maximize its technical efficiency score. The production frontier represents the maximum amounts of output that is produced by given amounts of input (the output maximization DEA model) or, alternatively, the minimum amounts of inputs required to produce the given amount of output (the input minimization DEA model).

Let us have *n* units that use *m* inputs to produce *r* outputs. The formulation of the input-oriented version of the constant returns-to-scale DEA model for unit *q* is:

2 $$\begin{aligned} {\text{Maximize}} \,\varphi_{q} & = \mathop \sum \limits_{k = 1}^{r} u_{k} y_{kq} \\ {\text{subject}}\, {\text{to}} & \mathop \sum \limits_{k = 1}^{r} u_{k} y_{kj} - \mathop \sum \limits_{i = 1}^{m} v_{i} x_{ij} \le 0, j = 1, 2, \ldots ., n, \\ \mathop \sum \limits_{i = 1}^{m} v_{i} x_{iq} & = 1, \\ u_{k} \ge \varepsilon , \,k & = 1, 2, \ldots ,r, \\ v_{i} \ge \varepsilon , \,i & = 1, 2, \ldots , m, \\ \end{aligned}$$

where *φ*_*q*_ is the technical efficiency score (the normalized capacity score of the region in this study), *x*_*ij*_ is the amount of input *i* used by unit *j*, *y*_*kj*_ is the amount of output *k* produced by unit *j*, and *ε* represents an infinitesimal constant that assures that the weight for each health resource is greater than zero. The output weights *u*_*k*_ and input weights *v*_*i*_ are variables in the model. In the input-oriented DEA model, the efficiency score *φ*_*q*_ is one if the unit *q* is technically efficient, and is lower than one if the unit is technically inefficient. The efficiency score calculates the size of input reduction that makes production unit *q* technically efficient. In the output-oriented DEA model, the efficiency score is one if the unit *q* is technically efficient, and is greater than one if the unit is technically inefficient.

The technical efficiency scores calculated from the input-oriented model with the regional population as input and the health resources as output expresses the lack of resources in comparison to the best-served regions that are represented by the set of efficient units. By using the efficiency scores, multiple health resources are now transformed into a single virtual resource, the amount of which is calculated as the regional population (POP_*j*·_) multiplied by the efficiency score *φ*_*j*·_ The production frontier method consists of three steps [27]:

1. For each region *j*, calculate the efficiency score *φ*_*j*_ by the input-oriented constant returns-to-scale DEA model with the regional population as input and health resources as outputs.
2. For each region *j*, calculate the value of virtual health resource VHR_*j*_ = *φ*_*j*_ × POP_*j*_.
3. Calculate the inequality measure for the virtual health resource.

### Inequality measures

Once the regional data on multiple health resources are expressed in the form of a single virtual resource by multiplying resource capacities by weights, the inequality measures can be applied. The measures of inequality express the variation in the observed variable by a single number. The simple measures of inequality are the absolute and relative ranges, decile ratios, coefficient of variation, etc. A very popular measure is the Gini coefficient, whose value is derived from the Lorenz curve, a cumulative frequency curve that compares the empirical distribution of the variable with the uniform distribution representing perfect equality. The value of the Gini coefficient ranges between 0, in the case of complete equality, and 1, in the case of complete inequality. Other well-known inequality measures are the Atkinson Index and the Theil Index. The Robin Hood Index (RHI) measures what proportion of resources has to be moved from regions with the above-average provision to regions with the below-average provision to achieve equal distribution. The RHI is calculated by the formula (3):

3 $${\text{RHI}} = \frac{1}{2}\mathop \sum \limits_{i = 1}^{n} \left| {\pi_{j} - \rho_{j} } \right|,$$

where *π*_*j*_ is the population proportion, *ρ*_*j*_ is the resource proportion, and *n* is the number of regions. The RHI is usually multiplied by 100 to be expressed in percentages.

There is available a variety of inequality measures described in the literature [28–30] and it is hard to say that one inequality measure is better than another. Kawachi and Kennedy [30] calculated the income distribution for the 50 U.S. states and studied the relation of income inequality to mortality. They used the Gini coefficient, the decile ratio, the proportions of total income earned by bottom 50, 60, and 70% of households, the Robin Hood Index, the Atkinson Index, and Theil’s entropy measure. All measures were highly correlated, and in no instance did the correlation coefficient fall below 0.86 in the absolute value, so there is little evidence to suggest that the choice of inequality measure will result in an absolutely different conclusion.

In order to measure the inequality, it is necessary to define what an appropriate geographical area is. A definition of geographical areas depends on the health resource the inequality of which is to be evaluated. The geographical areas are usually small for an analysis of outpatient services, relatively large for inpatient services, and very large for highly specialized services. It is hard to find information on such geographical areas as hospital service areas; therefore, the geographical areas that researchers analyse are states, provinces, regions, counties, districts.

### Data

In the Czech Republic, health services are reimbursed by public health insurance, which should guarantee equal access to health services for the whole population. The population coverage is virtually universal, and the range and depth of benefits available to insured individuals are broad. The health insurance system is financed through compulsory, wage-based contributions and through state contributions on behalf of economically inactive people, such as pensioners, children and the unemployed. Out-of-pocket payments remain among the lowest in OECD countries. Czech residents may freely choose their health insurance fund and healthcare providers. The health insurance funds must accept all applicants and no risk selection is permitted [31]. The Czech Republic is geographically a relatively homogeneous country.

The number of nurses and the number of physicians per 100,000 population are relatively similar to the EU average. The number of acute care hospital beds in the Czech Republic is still well above the EU average [31].

The country is administratively divided into 14 regions. Prague, the capital, has the status of a region. The region of Prague is located in the middle of the territory of the Středočeský region (Central Bohemia), and the population of the Středočeský region frequently uses health services in Prague. That is why the data of these two regions, the Prague and Středočeský regions, were joined together. The Czech regional data come from the Institute of Health Information and Statistics [32] and describe health resource capacities and population to December 31, 2017. These are the latest available data because the COVID-19 pandemic led to the postponement of the update of regional data.

The regional capacities of two health resources are evaluated: (1) the number of physicians in full-time equivalents (FTE) and (2) the number of nurses and midwives in FTE. In 2017, the Czech Republic had a population of 10.6 million inhabitants that were served by 49,562.1 physicians in FTE (4.68 per 1000 inhabitants) and by 84,076.4 nurses and midwives (7.94 per 1000 inhabitants). For the country where disease burden (health need) is distributed relatively evenly, regional population is the most straightforward criterion for allocating resources. The regional data are presented in Table 1.

**Table 1:** Regional data, Czech Republic, 2017

Region	Physicians in FTE (per 1000)	Nurses and midwives in FTE (per 1000)	Population
Prague/Středočeský	14,629.0 (5.56)	22,358.7 (8.49)	2,632,318
Jihočeský	2663.1 (4.17)	4704.4 (7.36)	639,180
Plzeňský	2732.6 (4.72)	4586.9 (7.92)	579,228
Karlovarský	1379.1 (4.66)	2411.1 (8.14)	296,106
Ústecký	2956.6 (3.60)	6225.3 (7.58)	820,937
Liberecký	1697.6 (3.85)	2695.2 (6.11)	440,934
Královéhradecký	2527.4 (4.59)	4854.9 (8.81)	550,848
Pardubický	2104.5 (4.07)	3562.0 (6.89)	517,243
Vysočina	2022.4 (3.98)	4029.4 (7.92)	508,664
Jihomoravský	6187.2 (5.24)	9847.6 (8.34)	1,180,477
Olomoucký	3061.9 (4.84)	5575.2 (8.81)	633,133
Zlínský	2324.7 (3.99)	4047.8 (6.94)	583,039
Moravskoslezský	5276.2 (4.37)	9178.3 (7.60)	1,207,419
Czech Republic	49,562.1 (4.68)	84,076.4 (7.94)	10,589,526

## Results

The results from all five models are presented in Table 2. In order to compare the results calculated by different models, the regional health resource capacity scores were normalized to the national value being 1. The separate evaluation of health resources shows that the joined region of Prague/Středočeský has the maximal number of physicians per 1000 inhabitants, which is 18.7% above the national average. The region of Královéhradecký has the maximum number of nurses and midwives, which is 11.0% above the national average. On the other hand, the minimal resource capacities are 77% of the national average for physicians (Ústecký) and 77% of the national average for nurses and midwives (Liberecký).

**Table 2:** Summary of models, results normalized to the national average (Czech Republic = 1)

Region	Separate evaluation (physicians)	Separate evaluation (nurses)	Expert model	Market model	Common weights	Production frontier
Prague/Středočeský	1.187	1.070	1.133	1.135	1.094	1.094
Jihočeský	0.890	0.927	0.907	0.907	0.920	0.920
Plzeňský	1.008	0.997	1.003	1.003	1.000	1.000
Karlovarský	0.995	1.026	1.009	1.009	1.019	1.019
Ústecký	0.770	0.955	0.855	0.853	0.917	0.941
Liberecký	0.823	0.770	0.798	0.799	0.781	0.781
Královéhradecký	0.980	1.110	1.040	1.039	1.084	1.094
Pardubický	0.869	0.867	0.868	0.868	0.868	0.868
Vysočina	0.849	0.998	0.918	0.916	0.968	0.983
Jihomoravský	1.120	1.051	1.088	1.089	1.065	1.065
Olomoucký	1.033	1.109	1.068	1.067	1.094	1.094
Zlínský	0.852	0.874	0.862	0.862	0.870	0.870
Moravskoslezský	0.934	0.957	0.945	0.944	0.953	0.953
Maximum	1.187	1.110	1.133	1.135	1.094	1.094
Minimum	0.770	0.770	0.798	0.799	0.781	0.781
Absolute range	0.418	0.340	0.335	0.336	0.313	0.313
Robin Hood Index (%)	6.24	3.60	4.98	4.96	4.10	3.99

In the expert model, we addressed former ministers of health, deputy ministers, hospital managers or deputy hospital managers, and professors of medicine with some managerial experience. In total, 23 experts were contacted by email, 12 of them responded. Only seven of them were able to give some value on substitution between physicians and nurses; two experts were not able to set the substitution rate and three experts did not agree with the possibility of substitution between physicians and nurses. The median value which will be used in the following calculation was such that one physician equals two nurses. In this case, the highest resource capacities are found in the Prague/Středočeský region (13.3% above the national average) and the lowest in the Liberecký region (20.2% below the national average).

A piece of valuable information was obtained from text comments in the questionnaires. One expert noted that in practice, substitution is a one-way relationship. One can substitute physicians by nurses, but not nurses by physicians. An economist can call it a one-way marginal rate of substitution. Some experts noted that substitution in practice is not possible due to legislation barrier and missing education programmes. Two experts noted that a missing doctor is a smaller problem than a missing nurse. On the labour market, nurses are more flexible than physicians and may easily leave the hospital or even the health care sector.

The resource weights in the market model were set as the ratio of average monthly salaries. In 2017, the average monthly salary of a Czech physician was 70,672 Czech korunas, and the salary of a nurse was 33,927 Czech korunas. The average salary of a physician was thus 2.08 times higher than the salary of a nurse. Using the average salaries as the weights, the values in Table 2 represent the total gross salary of physicians and nurses and midwives per inhabitant normalized to the national average. Again, the highest resource capacities are found in the Prague/Středočeský region (13.5% above the national average), and the lowest in the Liberecký region. The use of the market model is probably limited to human resources only. In the case of physical resources, the use of prices or costs may not make sense.

The optimal solution of the common weights model (1) gives weights 0.40 for physicians and 0.92 for nurses and midwives. Surprisingly, one physician is valued only as 0.43 nurse. The value of the objective function is 11.55, which was 88.8% of the theoretical maximum, which is 13. The scores in Table 2 are adjusted such that the Czech Republic as a whole scores 1. The problem of the common weights method lies in the fact that results are affected by the variability of resources. To maximize the value of the objective function, the model sets higher weights for the resources with smaller variability. This is unfortunately a serious disadvantage of the model because the lower weight for physicians can hardly be explained to health policy-makers. Hence, we advise using the common weight model with caution.

The production frontier was estimated by the input-oriented constant returns-to-scale DEA model (2) with the regional population as the single input and the number of physicians and the number of nurses and midwives as two outputs. In this model, the frontier is estimated by the most “inefficient” units, i.e. the regions with the maximal amount of resources. Three regions were technically efficient (Prague/Středočeský, Královéhradecký, Olomoucký) and formed the frontier that is 9.4% above the national value. The DEA scores are normalized such that the Czech Republic as a whole scores 1 (Table 2).

Finally, we used two measures of inequality, the absolute range and the Robin Hood Index, the advantage of which over other inequality measures is their simple health policy interpretation. The values of the absolute ranges were calculated directly from Table 2. Inequality in health resource capacities is lower by expert, market, common weights, and production frontier models than in the case of separate evaluation. In the case of Robin Hood Index, the results are not so strong, nevertheless, they show that inequality in the regional numbers of physicians is not so great if another resource (nurses and midwives) is taken into an account.

## Conclusion

The Czech Republic achieves a relatively high level of equity in financing. The majority of health services is financed by public health insurance, and the distribution of health resources is highly regulated. Health care coverage is comparatively extensive with an unusually broad range of health services being provided regardless of socioeconomic characteristics such as income or occupation [31]. Surprisingly, in spite of many regulations at the national and regional levels, we have found inequalities in regional resource distribution. This is a warning message for the Czech health policy-makers. According to Robin Hood Index, the recommendations for redistribution of resources vary between 3.60% and 6.24% (Table 2). However, these values are not so high.

The existence of unequal regional distribution of health resources and their unequal utilization have been described by research studies from many countries. The inequality measurement is a necessary tool that allows estimating the degree of inequality. Studies on regional inequalities in the distribution of health resources rarely analyse whether a region that has a smaller amount of one resource can be compensated by having a larger amount of another resource [e.g., 4]. We argue that as a first look at the data, a separate evaluation of regional inequalities of health resource capacities is a sensible approach, but it may be misleading. As a solution, we consider evaluating health resource capacities as a whole and consider the possibility of resource substitution. A comparison of the total resource capacity can be seen as a problem of multiple criteria decision-making in which the main issue is the setting of resource weights. By the resource weights, multiple regional health resources are transformed into a single virtual resource.

In this study, we have presented alternative methods of evaluation of regional health resource capacities: the separate evaluation, the expert model, the market model, the common weights model, and the production frontier model based on the data envelopment analysis. It is observed on the example of the Czech Republic that the models that take into account all health resources and the possibility of resource substitution show lower regional inequalities. From the view of health policy-makers, we advise using the common weight model with caution because the calculated values of weights can be in conflict with expectations and hardly explainable. The use of the market model is probably limited to human resources only. Health policy-makers will probably prefer to use an expert model, as they may have been involved in it, while the production model may be less comprehensible.

We are convinced that both researchers and policy-makers should always consider the possibility of resource substitutions in the assessment of regional variations in resource capacities. It also seems that the topic of this paper, the regional distribution of health workers, is closely related to the literature on skill-mix and task shifting and can also serve for discussion in this stream of research.

## Data Availability

Data are available from the Institute of Health Information and Statistics of the Czech Republic. Health care workers by occupation (full-time equivalents) 2010–2017. Prague. https://reporting.uzis.cz/cr/index.php?pg=statisticke-vystupy.
